# Use of television, videogames, and computer among children and adolescents in Italy

**DOI:** 10.1186/1471-2458-9-139

**Published:** 2009-05-13

**Authors:** Alessandro Patriarca, Gabriella Di Giuseppe, Luciana Albano, Paolo Marinelli, Italo F Angelillo

**Affiliations:** 1 Department of Public, Clinical and Preventive Medicine, Second University of Naples, Naples, Italy

## Abstract

**Background:**

This survey determined the practices about television (video inclusive), videogames, and computer use in children and adolescents in Italy.

**Methods:**

A self-administered anonymous questionnaire covered socio-demographics; behaviour about television, videogames, computer, and sports; parental control over television, videogames, and computer.

**Results:**

Overall, 54.1% and 61% always ate lunch or dinner in front of the television, 89.5% had a television in the bedroom while 52.5% of them always watched television there, and 49% indicated that parents controlled the content of what was watched on television. The overall mean length of time daily spent on television viewing (2.8 hours) and the frequency of watching for at least two hours per day (74.9%) were significantly associated with older age, always ate lunch or dinner while watching television, spent more time playing videogames and using computer. Those with parents from a lower socio-economic level were also more likely to spend more minutes viewing television. Two-thirds played videogames for 1.6 daily hours and more time was spent by those younger, males, with parents that do not control them, who watched more television, and who spent more time at the computer. The computer was used by 85% of the sample for 1.6 daily hours and those older, with a computer in the bedroom, with a higher number of computers in home, who view more television and play videogames were more likely to use the computer.

**Conclusion:**

Immediate and comprehensive actions are needed in order to diminish time spent at the television, videogames, and computer.

## Background

Public health and preventive campaigns targeted to early adolescence have mainly focused on reducing unhealthy behaviours such as physical and sport inactivity, eating patterns, television (TV) viewing, and videogame playing [1-3]. Indeed, a negative relationship exists between the amount of time spent watching TV and children and adolescents health status, including overweight [4,5], school and verbal performance [6,7], perceived cognitive and attention abilities [8,9], and violence or bullying [10,11]. The family structure is also likely to have an important influence on sedentary behaviours, and parental status, family size, and number of siblings may be related with higher levels of TV viewing.

The American Academy of Pediatrics has expressed concerns about the amount of time that children and adolescents spend viewing TV and has issued guidelines urging parents to limit total media time per day of quality programming, to remove TV from children's bedroom, and to monitor the shows children and adolescents are watching [12]. Similarly, *Physical Activity and Fitness Objectives in the Healthy People 2010 *has recommended that the proportion of students in grades 9 through 12 who view TV for two or more hours should be less than 25% [13]. In Italy, among adolescents aged 14–18 years 85.9% watched TV and 71.8% used internet at least three times a week [14], and the obesity prevalence rates was 5–6% in girls and 9% in boys aged 11–15 years [15] and 6% in boys and 5% in girls aged 11-year-old [16].

To the best of our knowledge, no previous epidemiologic survey has overall determined TV viewing, videogames playing, and computer use in children and adolescents in Italy. Thus, since it would be useful to obtain such data, the purposes of this study conducted in one region in Italy among a large sample of children and adolescents were: (a) to describe common practices about TV, videogames, and computer; (b) and to determine what association exist between these behaviours and different aspects of the family structure.

## Methods

The data for this cross-sectional survey were collected during the period between March and May 2007 from 1034 children and adolescents aged 11 to 16 years randomly selected from a random sample of 5 public schools in the geographic area of the Campania region, in the South of Italy.

Before the study, a meeting with the head of each school was arranged to present the study, and permission and collaboration were obtained. All parents of the selected students received an envelope with a letter informing them of the research project, describing the study, the voluntary nature of participation, and assurance of participation privacy and anonymity as no personal identifiers were included in the questionnaire. These policies were also printed explicitly on the front page of the questionnaire. Parent(s) provided the informed consent for their child participation before survey administration. The survey instrument was a self-administered anonymous structured questionnaire. On the day of the survey, in each classroom, a member of the research team gave oral instructions about filling in the questionnaire to the students who had secured parental consent. To preserve student privacy and to allow for anonymous participation, questionnaires were distributed and collected by a member of the research team, with no teacher involvement and no list of names or identifying information was created. Those who administered the questionnaire were advised only to respond to students' queries about the procedure and to guarantee the independent completion of the questionnaire.

The questionnaire collected information on the following items: socio-demographic characteristics; number of TVs and computers in the home, time spent on viewing TV (video inclusive), playing videogames, computer use, and performing sport activity; parental control over viewing TV, playing videogames, and using computer. Videogames playing indicated playing games on either consoles or computers, whereas computer use indicated any use other than games. Each participant was asked to indicate, in the "yes/no" format, if he/she views TV, plays videogames, and uses the computer. For each positive response, in order to assess the exposure, students were asked to indicate, in an open-ended format, the average daily amount of time they spent either in the home or elsewhere. In addition, for each meal or snack, the children were asked whether they participated in any of the following activities while eating: watching TV, playing videogames, or using the computer. Each question was measured on a five-point Likert scale and the possible responses were "never", "rarely", "sometimes", "often", and "always". The first four categories regarding TV for the questions about lunch and dinner were grouped into not always eat during TV viewing. The students were also asked to indicate, in an open-ended format, the average weekly amount of time of performing sports activity. This variable was thereafter transformed into minutes per day of performing sports activity. Finally, each student was asked to indicate, in the "yes/no" format, if he/she has a TV and a computer in the bedroom and if the parent(s) control or supervise TV watching, playing videogames, and using the computer.

Health care professionals measured in the classroom height and weight with digital scales (weight to the nearest 0.1 kg) and a portable stadiometer (height to the nearest 0.1 cm) while the children were not wearing shoes. The body mass index (BMI) of each child was calculated as weight in kilograms divided by height in meters squared (kg/m^2^) and internationally accepted gender-specific and age-specific cut-off points for BMI were adopted to categorise children as overweight or obese [17].

Prior to study commencement, a pilot survey was conducted to test question formats and sequence to an appropriate cognitive and reading level.

The study protocol was reviewed and approved by the Ethics Committee of the Second University of Naples.

### Statistical analysis

Multiple logistic and linear regression analyses were used. Four models were developed including those variables that were considered to be potentially associated with the following outcomes of interest: viewing TV for at least two hours per day (Model 1); mean minutes per day of TV viewing (Model 2); mean minutes per day of videogames playing (Model 3); mean minutes per day of computer using (Model 4). The following explanatory variables were included in all models: age (continuous), gender (0 = male, 1 = female), number of siblings (0 = 0, 1 = 1, 2 = 2, 3 =≥ 3), both parents in the household (0 = no, 1 = yes), parent's working activity as a two dummy variables with unemployed as the reference group (lower managerial, artisans, commercial: 0 = no, 1 = yes; high professional, managerial: 0 = no, 1 = yes), and mean minutes per day of performing sport activity (continuous). These other predictor variables were included: in Models 1 and 2, number of TVs in the home (continuous), routinely viewing TV in the bedroom (no = 0, yes = 1), always eat lunch or dinner during TV viewing (no = 0, yes = 1), TV in the bedroom (no = 0, yes = 1), and parental control in viewing TV (no = 0, yes = 1); in Models 3 and 4, mean minutes per day of TV viewing (continuous); in Models 1 to 3, mean minutes per day of computer using (continuous); in Models 1, 2, and 4, mean minutes per day of videogames playing (continuous); in Model 3, playing videogames not with somebody else (no = 0, yes = 1), and parental control in playing videogames (no = 0, yes = 1); in Model 4, number of computer in the home (continuous), computer in the bedroom (no = 0, yes = 1), parental control in computer using (no = 0, yes = 1), and computer use to play (no = 0, yes = 1). In the logistic regression models the adjusted Odds Ratios (ORs) and the corresponding 95% confidence intervals (CIs) were calculated for each independent variable. All statistical tests were two-sided and a *p*-value ≤ 0.05 was considered to be statistically significant. All statistical procedures were performed by using Stata software (Version 10) [18].

## Results

Of the 1034 questionnaires distributed, 47 were excluded due to inconsistent information because the participants reported, for example, not playing videogames but he/she indicated the amount of time of playing per day. A total of 987 questionnaires were considered for a response rate of 95.4%. A description of the general characteristics of the study population is provided in Table 1. The mean age was 13.7 years, more than half were males, almost all lived with the parents, the mean BMI was 23.1, and 22.8% were classified as overweight (including obese).

**Table 1:** Characteristics of the study population

	**N**	**%**
***Gender***		
Male	511	51.8
Female	476	48.2
***Age (years)***	13.7 ± 1.4*	
***Both parents in the household***		
Yes	924	93.6
No	63	6.4
***Number of siblings***		
0	97	9.8
1	497	50.4
2	286	29
≥ 3	107	10.8
***Parent's working activity***		
Unemployed	34	3.4
Lower managerial, artisans, commercial	832	84.3
High professional, managerial	121	12.3
***Height (m)***	1.65 ± 0.1 (1.35–1.91)*	
***Weight (kg)***	63.2 ± 15.1 (26–125)*	
***Body Mass Index (BMI)***	23.1 ± 4.2 (14.1–40.7)*	
***Overweight (including obesity)***		
Yes	225	22.8
No	762	77.2
***Performing sport activity (minutes per day)***	30.2 ± 32.3 (0–205.7)*	

*Mean ± Standard deviation (range)

The self-identified practices regarding TV, videogames, and computer of the study participants are presented in Table 2. Overall, 58.4% always ate lunch or dinner in front of the TV, 89.5% had a TV in the bedroom while 52.5% of them always watched the TV there, 49.3% indicated that parents controlled the content of what was watched, the vast majority (74.9%) watched the TV at least two hours per day, and more than half had a computer in the bedroom.

**Table 2:** Time spent on TV viewing, videogames playing, and computer using in the study population

	**N**	**%**
***Number of TVs in the home***		
1	18	1.8
2	140	14.2
3	452	45.8
4	267	27.1
> 4	110	11.1
***TV in the bedroom***		
Yes	883	89.5
***Parental control in viewing TV***		
Yes	487	49.3
***At least two hours per day of watching TV***		
Yes	739	74.9
***TV viewing (minutes per day)***	170 ± 76 (26–386)*	
***TV viewing in the bedroom***^+^		
Yes	464	52.5
***Always eat lunch or dinner while watching TV***		
Yes	576	58.4
***Videogames playing***		
Yes	588	59.6
***Videogames playing (minutes per day)***^++^	99 ± 65 (8–360)*	
***Parental control in playing videogames***^++^		
Yes	188	32
***Playing videogames not with somebody else***^++^		
Yes	130	22.1
***Computer using***		
Yes	835	84.6
***Number of computers in the home***^+++^		
0	15	1.8
1	658	78.8
2	129	15.5
≥ 3	33	3.9
***Computer in the bedroom***^+++^		
Yes	512	61.3
***Computer using (minutes per day)***^+++^	97 ± 65 (8–360)*	
***Parental control in computer using***^++^		
Yes	485	58.1
***Using computer to play***^+++^		
Yes	88	10.5

* Mean±Standard deviation (range)

+ Only for those who have a TV in the bedroom

++ Only gamers

+++ Only computer users

Table 3 detailed the results of the multivariable logistic and linear regression models created to determine the relative contribution of each predictor when examined simultaneously on the different outcomes. Model 1 presents the cumulative ORs from multivariate logistic regression modelling used to explore the relationship between several variables and watching the TV at least two hours per day. Those older (OR = 1.16; 95% CI 1.04–1.3), who always ate lunch or dinner while watching TV (OR = 1.82; 95% CI 1.32–2.52), who spent more time playing videogames (OR = 1.006; 95% CI 1.003–1.008) and using computer (OR = 1.006; 95% CI 1.003–1.009) were more likely to watch the TV at least two hours per day. Overall, the mean length of time spent viewing TV was 2.8 hours per day and the multivariate linear adjustments indicated that the factors associated with this outcome were similar to those associated with watching the TV at least two hours per day. In addition, respondents with lower managerial, artisans, or commercial parents spent significantly more minutes per day on TV viewing (Model 2). Almost two-thirds played videogames (59.6%) for 1.6 hours per day and the multivariate linear regression analysis reported that this amount was significantly higher in those younger, males, who watched more TV, who spent more time at the computer, and when the parents do not control when they play videogames (Model 3). Finally, 85% stated that they used the computer for an average of 1.6 hours per day. Multiple linear regression model indicated that the adjusted daily mean minutes of computer using was significantly higher in those older, who had a computer in the bedroom, who had a higher number of computer in the home, who view more TV, and who play more time with videogames (Model 4).

**Table 3:** Multivariate logistic (1) and linear (2–4) regression analyses indicating associations between several variables and the different outcomes

Variable	OR	95% CI	*p*
**Model 1**. Viewing TV for at least two hours per day			

Log likelihood = -518.81, χ^2 ^= 75.16 (14 df), *p *< 0.0001			

Age	1.16	1.04–1.3	0.006

Gender	1.22	0.86–1.73	0.26

Number of siblings	0.98	0.81–1.18	0.83

Both parents in the household	0.86	0.44–1.68	0.67

Parent's working activity as lower managerial, artisans, commercial	1.4	0.61–3.19	0.43

Parent's working activity as high professional, managerial	1.1	0.44–2.74	0.84

Number of TVs in the home	0.97	0.82–1.16	0.77

TV in the bedroom	0.85	0.5–1.46	0.57

Routinely viewing TV in the bedroom	1.05	0.76–1.44	0.78

Parental control in viewing TV	0.93	0.68–1.27	0.66

Always eat lunch or dinner during TV viewing	1.82	1.32–2.52	< 0.001

Mean minutes per day of performing sport activity	0.99	0.99–1.001	0.69

Mean minutes per day of computer using	1.006	1.003–1.009	< 0.001

Mean minutes per day of videogames playing	1.006	1.003–1.008	< 0.001

Variable	Coeff.	t	*p*

**Model 2**. Mean minutes per day of TV viewing			

F(14,972) = 7.23, *p *< 0.0001, R^2 ^= 9.4%, adjusted R^2 ^= 8.1%			

Age	5.09	2.96	0.003

Gender	-0.68	-0.12	0.9

Number of siblings	3.8	1.31	0.19

Both parents in the household	-4.98	-0.51	0.61

Parent's working activity as lower managerial, artisans, commercial	28.7	2.2	0.03

Parent's working activity as high professional, managerial	25.67	1.77	0.08

Number of TVs in the home	-0.4	-0.15	0.88

TV in the bedroom	1.23	0.15	0.89

Routinely viewing TV in the bedroom	-7.88	-1.58	0.12

Parental control in viewing TV	0.04	0.01	0.99

Always eat lunch or dinner during TV viewing	23.65	4.86	< 0.001

Mean minutes per day of performing sport activity	-0.01	-1.25	0.21

Mean minutes per day of computer using	0.14	4.02	< 0.001

Mean minutes per day of videogames playing	0.18	4.5	< 0.001

Variable	Coeff.	t	*p*

**Model 3**. Mean minutes per day of videogames playing			

F(11,576) = 8.58, *p *< 0.0001, R^2 ^= 14.1%, adjusted R^2 ^= 12.4%			

Age	-7.85	-4.53	< 0.001

Gender	-35.11	-6.13	< 0.001

Number of siblings	-2.61	-0.86	0.39

Both parents in the household	-13.64	-1.4	0.16

Parent's working activity as lower managerial, artisans, commercial	-7.44	-0.5	0.62

Parent's working activity as high professional, managerial	-16.01	-0.98	0.33

Parental control in playing videogames	-12.11	-2.21	0.028

Playing videogames alone	-2.74	0.45	0.65

Mean minutes per day of performing sport activity	-0.009	-0.85	0.4

Mean minutes per day of computer using	0.09	2.47	0.014

Mean minutes per day of TV viewing	0.15	4.58	< 0.001

Variable	Coeff.	t	*p*

**Model 4**. Mean minutes per day of computer using			

F(13,821) = 5.9, *p *< 0.0001, R^2 ^= 8.5%, adjusted R^2 ^= 7.1%			

Age	3.4	2.1	0.04

Gender	0.72	0.14	0.89

Number of siblings	-3.29	-1.22	0.22

Both parents in the household	5.96	0.67	0.51

Parent's working activity as lower managerial, artisans, commercial	-2.71	-0.2	0.84

Parent's working activity as high professional, managerial	-4.53	-0.38	0.71

Number of computer in the home	16.62	4.07	< 0.001

Computer in the bedroom	10.36	2.27	0.02

Parental control in computer using	1.46	0.32	0.75

Computer use to play	-11.32	-1.55	0.12

Mean minutes per day of performing sport activity	0.002	0.25	0.8

Mean minutes per day of videogames playing	0.12	3.2	0.001

Mean minutes per day of TV viewing	0.13	4.45	< 0.001

## Discussion

This study seeks to assess common practices about TV viewing, videogames playing, and computer using and also to identify which variables are associated with these behaviours among a large sample of children and adolescents 11- to 16-year-olds in one region in Italy.

It is important to emphasize that the survey responses indicate an inordinately high amount of time spent in viewing TV with an exposure of 2.8 hours per day. Early studies showed similar values with 3.1 hours in children aged 12 to 13 in the United States [19], 3.13 hours per weekday in 13 and 15 years in New Zealand [20], and 3–3.57 in girls and boys with a mean age of 13 years in Belgium [21]. However, substantially lower exposure was reported with 1.9 hours per day in boys and girls aged 15–16 from three regions in Europe [22], 1.91 in girls at age 11 in the United States [4], and 2.2 in children 11–14 years old in France [23]. International comparisons, however, must be taken with caution mainly because of differences across the studies such as, for example, the characteristics of the sample and the study's methodology. Multivariate analyses in this study provided evidence of strong interrelations among several behaviours: the frequency of TV viewing was positively associated with the increased frequency of playing videogames and using computer. This finding is consistent with other surveys [4,24]. Approximately 75% of the sample spent at least two hours per day in watching the TV. Similar values have been observed in the United States in youth aged 14–16 years, although measured only on school-day, ranging from 67% to 77.3% in boys and 65.8% to 67.3% in girls [25], and from 68% and 78% in children of 11–13 and 14–16 years [26]. The value was considerably higher than the prevalence of 37.7% to 51% in 14–16 years [27] and 56.4% and 66.9% in 11–13 and 14–16 years [28]. Age was a significant predictor of this behaviour, since the frequency of those who watched TV ≥ 2 hours per day was significantly higher in later childhood. Moreover, an association has been observed between parental working activity, an indicator of socio-economic status, and amount of TV watching per day, with respondents with lower managerial, artisans, or commercial parents spending more minutes.

A disturbing discovery was the large percentage (89%) who acknowledged the presence of a TV in the bedroom, although no evidence was found to link this variable with the daily amount of time viewing TV. In previously cross-sectional studies, lower prevalence were observed with values of 49.8% and 55.2% in children aged 11–12 years in the United States [29] and of 23.5% and 35% in the already mentioned Belgium study [21]. Another disturbing discovery was the frequency in which parents consistently monitor their children's TV viewing (49%), videogames playing (32%), and computer using (58.1%), but also in this case there was no significant relationship between amount of TV viewing and parental control. Moreover, the multivariate analysis showed that in only one model the supervision has an important role, because children play videogames a significant higher amount of time if the parents do not control when they play.

With regard to videogames playing and computer using, in the current study the mean daily usage was 1.6 hours for both. These findings are consistent with the results from the United States on similarly aged children with a mean time spent daily of videogames playing and computer using of 1.49 and 1.19 hours, respectively [19]. In Belgium, the daily time spent on videogames playing was 54 minutes among boys [21]. Multivariate models revealed that the amount of times exposed to videogames and computer were related to the TV viewing with both that increase as TV view increases. Furthermore, respondents who spent more time on videogames playing were more likely to be males and younger, whereas those who spent more time using computer were older.

Some potential limitations of the present study need to be acknowledged. First, the observational nature does not permit definitive establishment of a causal relationship between predictors and outcomes. The data reported are interesting with respect to the magnitude and the direction of the different relationship, but they cannot untangle the direction. For example, it is not possible to assume that if parents control their children when they play videogames they would be spending less time playing. Although this seems reasonable, it remains to be verified. Moreover, although in the multivariate analysis we have attempted to control for theoretically relevant predictors of the outcomes, it is possible that there are other unmeasured potential confounding variables, such as other family, social, and environmental characteristics, that influence the outcomes. Second, the methodology used for the quantification of watching TV, playing videogames, or using computer was self-report by the respondents. Participants were assured that their responses would not be shared with others and anonymity while answering the questionnaire may guarantee honest and more accurate self-report. Nonetheless, it was likely that existed the possibility that students may either over-report or under-report information about the media time. Distortions motivated by the desire to please and reluctance to tell the true might also be present. However, it has been proved that a self-administered questionnaire is valid for group comparisons regarding watching TV, playing videogames, or using computer [24]. Despite these limitations, it should be recognized that an important strength of the study is the very high participation rate which allows many comparisons providing representative and generalizability information from a large and heterogeneous sample of adolescents in one region in Italy and to extend the perspective research on different factors associated with watching TV, playing videogames, or using the computer.

## Conclusion

These results extend the understanding about TV viewing, videogames playing, and computer using and their relationships with different variables in children and adolescents and immediate and comprehensive actions are needed in order to diminish time expended at the TV, videogames, and computer.

## Competing interests

The authors declare that they have no competing interests.

## Authors' contributions

AP participated in the design of the study, collected the data, and contributed to the data analysis; GDG collected the data, and contributed to the data analysis and interpretation; LA participated in the design of the study, and contributed to the data analysis and interpretation; PM participated in the design of the study and contributed to the interpretation of the data; IFA, the principal investigator, designed the study, was responsible for the data collection, statistical analysis and interpretation, and wrote the article. All authors have read and approved the final version of the manuscript.

## Pre-publication history

The pre-publication history for this paper can be accessed here:


